# Innate immunity in the Grid2^*Lc/+*^ mouse model of cerebellar neurodegeneration: glial CD95/CD95L plays a non-apoptotic role in persistent neuron loss-associated inflammatory reactions in the cerebellum

**DOI:** 10.1186/1742-2094-10-65

**Published:** 2013-05-15

**Authors:** Béatrice Vernet-der Garabedian, Paul Derer, Yannick Bailly, Jean Mariani

**Affiliations:** 1 Université Pierre et Marie Curie-P6, UMR 7102, Paris F75005, France; 2 CNRS UMR7102, Paris F75005, France; 3 Hôpital Charles Foix, Institut de la Longévité, Ivry-sur-Seine, 94205, France; 4 UPR 2356 CNRS, Strasbourg, France

**Keywords:** Glial reaction, Astrocyte, Microglia, Cytokines, GFAP, IL-6, Soluble CD95L, Lurcher, Grid2 gene

## Abstract

**Background:**

There is growing evidence that the death receptor CD95 has a wider role in non-apoptotic functions. In the brain, it may contribute to neural death and to the associated inflammatory reaction via a non-apoptotic pathway. Brain injury triggers an inflammatory reaction in which the CD95/CD95L system acts principally through peripheral cells recruited to the lesion. In cases of inflammation within the brain, with no blood–brain barrier leakage, the role of the CD95/CD95L system is thus unclear. We investigated the possible role of CD95 and CD95L in such conditions, by studying the relationships between glial cell activation, neuron death and CD95/CD95L expression in the cerebellum of the Lurcher (Grid2^*Lc/+*^) mutant mouse, a model of cerebellar neurodegeneration.

**Methods:**

Glial cells in slices of wild-type and *Lurcher* mouse cerebella were observed by light microscopy at various ages overlapping periods of neuron loss and of pre- and post-neurodegeneration. Subcellular organization was studied by electron microscopy. We assessed CD95 levels by western blotting, RT-PCR and glial cell cultures. The levels of CD95L and IL-6 were studied by ELISA and a biological assay, respectively.

**Results:**

In the Grid2^*Lc/+*^cerebellum, neuron loss triggers a typical, but abnormally persistent, inflammatory reaction. We identified two phases of astrogliosis: an early burst of large glial cell activation, peaking at postnatal days 25 to 26, coinciding with peak cerebellar neuron loss, followed by a long period of slow decline indicating that the strength of the glial reaction is modulated by neuron mortality rates. Comparisons of time-courses of glial cell activation, cytokine production and neuron loss revealed that the number of surviving neurons decreased as CD95 increased. Thus, CD95 cannot be directly involved in neuron death, and its role must be limited to a contribution to the inflammatory reaction. The upregulation of CD95 likely on astrocytes coincides with increases in the levels of IL-6, a cytokine produced principally by astrocytes, and soluble CD95L.

**Conclusions:**

These results suggest that CD95 and soluble CD95L contribute, via non-apoptotic signaling, to the inflammatory reaction initiated early in neuron death within the Grid2^*Lc/+*^ cerebellum.

## Background

Central nervous system (CNS) inflammation may develop in response to brain injury or in association with neurodegeneration in human neurodegenerative diseases, such as Parkinson’s, Huntington’s and Alzheimer’s diseases. Inflammation resulting from trauma or ischemia reperfusion injury includes endothelial damage, the release of proinflammatory mediators, changes in vascular permeability, infiltration with peripheral inflammatory cells and the activation of microglia, the innate immune cells of the brain, and astrocytes. It is marked by the recruitment of peripheral blood cells and the formation of a scar. CD95 (Fas, APO-1), which was initially identified as a death receptor, and its cognate ligand (CD95L, Fas-L) are both expressed in the brain. They are thought to contribute to the inflammatory reaction through non-apoptotic signaling, mediating proinflammatory responses and triggering the recruitment of peripheral macrophages and leukocytes to the site of inflammation [1,2]. Recent data obtained by Letellier *et al.*[3] for the spinal cord lesion model indicate that the expression of CD95L on peripheral myeloid cells plays a crucial role in the neural cell reaction at the lesion. These authors demonstrated that peripheral cell CD95L expression, which is upregulated by the lesion, is required for the recruitment of peripheral myeloid cells to the injured tissue and contributes to neural cell loss by acting on peripheral myeloid cells, rather than via direct CD95-induced apoptosis in CD95-bearing resident neural cells [3]. In neurodegenerative diseases, the glial cell activation associated with neurodegeneration is chronic. The inflammatory reaction is mild and features an absence of leukocytes within the neural parenchyma tissue; the possible occurrence of peripheral monocyte infiltration remains a matter of debate [4-6]. Despite these important differences between the two types of inflammatory reaction, the CD95/CD95L system has long been considered to play similar roles in the two situations [1,7]. However, in the absence of infiltrating peripheral cells, the contribution of the CD95/CD95L system to neural cell reactions (neural degeneration and the glial cell inflammatory reaction) remains unclear. Natural mutant mice, such as the ataxic Lurcher (Grid2^*Lc/+*^) mouse, which displays cerebellar neurodegeneration, are valuable for investigations of the role of the CD95/CD95L system in this context making use of our extensive knowledge of cerebellar neurodegeneration in this mutant [8].

Ataxia becomes clinically detectable in the Lurcher mouse at about postnatal day 15 (P15). It results from the progressive postnatal loss of cerebellar neurons due to a mutation in the ∂2 glutamate receptor subunit (GluR∂2) gene (*Grid2*). The Lurcher mutation is a semi-dominant gain-of-function mutation involving a base-pair change [9]. Within the cerebellum, GluR∂2 is present specifically in Purkinje cells [10,11]. The expression of the mutated allele leads to a cell-autonomous process of Purkinje cell degeneration occurring between P7 and P30 [8]. As a result, 90% of the granule cells and 60 to 75% of the inferior olivary neurons die within the first 3 months after birth, due to the loss of their target [10,11]. The mechanisms by which Purkinje cells die have remained elusive. Activated caspase-3, DNA fragmentation and autophagosomes in dying Purkinje cells were thought to be the results of the activation of various cell-death execution pathways [12-14]. Recent *in vitro* and *in vivo* studies supported a necrosis mechanism caused by excitotoxicity and accompanied with features of autophagy [15]. In the Grid2^*Lc/+*^ cerebellum, this degenerative process takes place in an intact brain with a fully functional blood–brain barrier (N Delhaye-Bouchaud and J Mariani, unpublished data).

The glial reaction to neuron loss has remained largely unexplored in the Grid2^*Lc/+*^ cerebellum. The presence of reactive astrocytes and high IL-1ß concentrations reported in previous studies suggest that an inflammatory process occurs in the Grid2^*Lc/+*^ cerebellum [16,17].

In this study, we investigated the possible role of the CD95/CD95L system in the mechanism of neuronal death and in the inflammatory response, as an intrinsic component of the glial reaction in the Grid2^*Lc/+*^ cerebellum. We analyzed the relationship between the death of cerebellar neurons and glial activation. Correlations between glial cell activation and CD95 and CD95L expression on the one hand, and neurodegeneration events occurring from birth into adulthood on the other, indicate that the CD95/CD95L system is involved in the inflammatory reaction, mostly through astrocytes.

## Materials and methods

### Animals

Heterozygous Grid2^*Lc/+*^ mutant mice (Lc/+) and the corresponding wild-type strain, C57BL/6J X CBA (+/+), were obtained by crossing Lc/+ males with +/+ females. Mutant offspring were identified on the basis of ataxic behavior at about P15, or by genotyping for younger animals, as previously described [12]. Animals were cared for and killed in accordance with the guidelines of the French National Ethics Committee for Life and Health Sciences.

### Glial cell cultures

High-purity cultures of astrocytes and microglia were prepared from mice from 5- and 1-day-old Lurcher litters, respectively, as previously described [18].

### Tissue collection

Cerebella and cerebral cortices were collected on ice in an RPMI bath. The visible meninges and vessels were stripped away under a binocular microscope [17]. The dissected tissue was then immediately frozen in liquid nitrogen and stored at -80°C until use.

### Immunoblotting

We homogenized 50 to 80 mg tissue in an Ultra-Turrax homogenizer in lysis buffer containing 50 mM Tris, 150 mM NaCl, 0.02% sodium azide, 1% Igepal NP-40 and protease inhibitors (100 μg/ml 4-(2-Aminoethyl) benzenesulfonyl fluoride hydrochloride (AEBSF), 2 μg/ml iodoacetamide, 2 μg/ml leupeptin, 10 μg/ml soybean trypsin inhibitor (SBTI), 2 μg/ml pepstatin, 1 μg/ml aprotinin). Lysates were then centrifuged (10500 × *g* for 30 minutes) and the supernatants were collected. Total protein concentrations were determined on triplicate samples with the DC Protein Assay from BIO-RAD, based on the Lowry method. Samples were heated at 100°C for 1 minute in loading buffer (125 mM Tris HCl, pH 6.8, 4% SDS, 0.025% bromophenol blue, 20% glycerol, 10% 2-mercaptoethanol). Proteins from 30 μg brain extract were separated by SDS-PAGE in a 12% acrylamide gel and transferred to Hybond-C super nitrocellulose membranes (Amersham, Buckingham, UK). Membranes were blocked by incubation with 5% non-fat milk powder and 5% normal goat serum in TBS and incubated overnight at 4°C with 0.2 μg/ml rabbit polyclonal M-20 anti-Fas antibody (Santa Cruz Biotechnology) in TBS supplemented with 0.2% gelatin. The immunocomplex was detected with peroxidase-conjugated anti-rabbit secondary antibodies (Jackson ImmunoResearch Laboratories, Inc.), with diaminobenzidine or a chemiluminescent substrate (Amersham Pharmacia Biotec.).

### Immunohistochemistry

Mice were anesthetized by an intraperitoneal injection of 3.5% chloral hydrate (0.1 ml/g body weight) and transcardially perfused with buffer A (0.1 M cacodylate buffer pH 7.2, containing 4% paraformaldehyde, 8% saccharose and 5% dimethylsulfoxide). Brains were postfixed by overnight incubation in buffer A at 4°C and rinsed by incubation for 1 day with buffer A without dimethylsulfoxide. A vibratome (LEICA VT 1000S) was then used to cut one hemisphere into 30 μm sagittal sections, which were placed in 0.2 M Tris-maleate buffer (pH 7.2) supplemented with 8% saccharose. Microglia were detected and stained by the nucleoside diphosphatase histo-enzymological method, as previously described [19]. Briefly, sections were incubated with 2 mM 5′-uridine-diphosphate in 80 mM Tris-maleate buffer (pH 7.2) supplemented with 0.12% lead citrate, 5 mM manganese chloride and 1% dimethyl sulfoxide for 30 minutes at 37°C. The reaction product was detected as a brown precipitate by incubating sections in 2% ammonium sulfide. For glial fibrillary acidic protein (GFAP) labeling, sections prepared as described above were washed three times in TBS and incubated in TBS supplemented with 1% Triton X-100 and 10% normal goat serum for 20 minutes. Rabbit polyclonal anti-GFAP antibody (Sigma; 1/200 dilution in TBS), and biotinylated goat anti-rabbit IgG antibody (affinity-purified, from Vector Laboratories; 1/500 dilution), were applied to the sections, which were then incubated overnight at 4°C and for 3 hours at room temperature, respectively. Labeling was visualized with the Vectastain ABC kit, using diaminobenzidine (Sigma) as the chromogen.

### Electron microscopy

Two Grid2^*Lc/+*^ mice were anesthetized with sodium pentobarbital (15 mg/50 g body weight, Sanofi Diagnostic Pasteur) and fixed by transcardial perfusion with 300 ml 1% paraformaldehyde and 1% glutaraldehyde in 0.1 M phosphate buffer (pH 7.3). Sagittal sections (1 mm) of the cerebellar vermis were cut with a vibratome and postfixed by incubation for 1 hour in 1% osmium tetroxide. They were then dehydraded by passage through a series of concentrations of ethanol and embedded in Araldite. Ultrathin sections (60 nm thick) cut from blocks (1 mm^3^) sampled from the vibratome sections were counterstained with uranyl acetate and examined with a Hitachi 7500 transmission electron microscope.

### Semiquantitative RT-PCR

Total RNA was isolated from frozen brain tissues with the RNeasy Mini kit (Qiagen, Hilden, Germany) and from cultured cells with Trizol reagent. RNA concentration and purity were determined with a Nanodrop ND-1000 spectrophotometer. The reverse transcription reaction was performed with a Reverse Transcription System kit (Promega), with avian myeloblastosis virus transcriptase and oligo(dT)15 primers, according to the manufacturer’s instructions. The first-strand cDNA was amplified by PCR in a Perkin Elmer Thermal Cycler, with *Taq* polymerase and the primers for GFAP (sense primer TTC CTG TAC AGA CTT TCT CC and antisense primer CCC TTC AGG ACT GCC TTA GT); Fas (sense primer CAT CTC CGA GAT TTT AAA GC and antisense primer GTT TCC TGC AGT TTG TAT TGC T) and hypoxanthine-guanine phosphoribosyltransferase (HPRT) (sense primer CCT GCT GGA TTA CAT TAA AGC ACT G and antisense primer GTC AAG GGC ATA TCC AAC AAC AAA C). We used appropriate concentrations of cDNA to ensure that PCR was carried out in non-saturated conditions. Amplification products were visualized by electrophoresis in a 2% agarose gel containing ethidium bromide (0.6 mg/ml), and quantified by densitometry with Densylab software (Microvision Instruments, Evry, France). Values were normalized with respect to the amplicon for the *HPRT* housekeeping gene. A stimulation index (SI) was calculated for the kinetic study. SI is defined as the normalized CD95 mRNA level for stimulated cells from which we subtracted the normalized CD95 mRNA level for unstimulated cells, before dividing the result by the normalized CD95 mRNA level for unstimulated cells.

### Determination of cytokine levels

IL-6 activity and CD95L concentration were determined on tissue extracts prepared as previously described [17]. Briefly, frozen tissues were rapidly weighed, dissociated with a Dounce Potter homogenizer on ice with five parts by weight of RPMI containing antiproteases (10 mM amino-*n*-caproic acid, 0.5 mM benzamidine, 1 mM ethylenediaminetetraacetic acid (EDTA) and 20 μM AEBSF for CD95L and only EDTA and AEBSF for IL-6). The resulting homogenate was centrifuged for 30 minutes at 13400 × *g* at 4°C. Supernatants were collected, and small aliquots were immediately frozen at -20°C until use. Total protein concentrations were determined as described above for immunoblotting. Two cerebella from different animals were used to prepare a single extract. Each sample was analyzed in duplicate. IL-6 activity was determined in a biological assay with the IL-6-dependent B-cell hybridoma B9. B9 cells (5 × 10 ^3^ cells/well, in 100 μl) were plated in 96-well microtiter plates, and 100 μl of a serial dilution of extract and murine IL-6 as a standard were added. The plates were incubated for 72 hours at 37°C in a humidified chamber containing 5% CO_2_-95% air. B9 cell growth was then assessed in an MTT assay. We incubated 0.5 mg/ml MTT with cells for 3.5 hours in a culture incubator. Insoluble purple formazan salts were solubilized with 150 μl isopropanol plus 0.1 M HCl and absorbance was determined at 560 nm. Fas-L concentrations were measured in a sandwich ELISA with the Quantikine M kit from R&D Systems, in accordance with the manufacturer’s instructions. Experiments were repeated two or three times.

### Statistical analysis

Group difference was established by using the non-parametric Mann Whitney test or with a one-way analysis of variance with de Scheffé multiple comparison test for multiple group comparison. Homogeneity of the variance was assessed using F-test. Statistical analyses were carried out using StatView software (Abacus Concept).

## Results

Early neurodegeneration in the Grid2^*Lc/+*^ cerebellum is associated with the persistent activation of glial cells.

**Figure 1:**
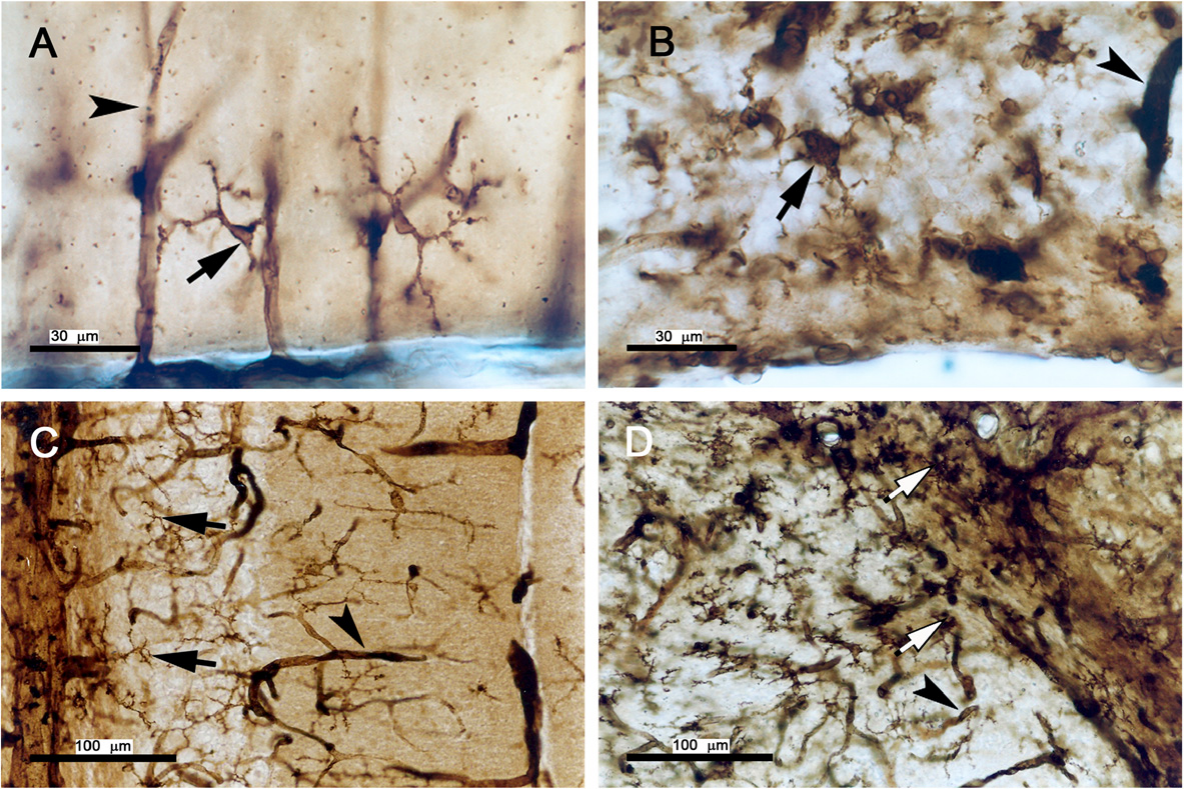
**Microglial activation in the Grid2**^***Lc/+ ***^**cerebellum. **Sagittal sections of wild-type **(A,C) **and Grid2^*Lc/+ *^**(B,D) **cerebella from 21-day-old (A,B) and adult (C,D) mice were stained by the dinucleoside diphosphatase technique. Microglia (arrow) and blood vessels (arrow-head) are labeled. (B and D) Isolated activated microglial cells with enlarged cell bodies and thick processes are present specifically in the cerebellum of 21-day-old Grid2^*Lc/+ *^mice (B). In adult (D) mice, microglia are mostly organized into clusters (open arrow). Scale bars: A and B, 30 μm; C and D, 100 μm.

Grid2^*Lc/+*^ cerebellar neurons die between P7 and P90, with large-scale degeneration occurring between P17 and P25 [11]. We studied the time course of the glial cell reaction to neuron loss by following glial morphological changes from P5 to adulthood by GFAP immunolabeling for astrocytes and the histoenzymatic nucleoside diphosphatase method for microglia. Cerebella from P5, P13, P21 and P150 mice were studied. At P5, round or ameboid microglial cells were present in the cerebellar white matter of both wild-type and mutant mice (data not shown). These microglia were of the macrophage type and were subsequently replaced by microglia displaying signs of activation, with an enlarged cell body and short thick processes, in the Grid2^*Lc/+*^ cerebellum, and by non-activated microglia in the wild type (see Figures 1A and 1B, respectively). At P13, activated microglia were detected either as isolated cells or in sparse clusters, indicating the occurrence of a microglial activation process that began before this time point. Clusters were first observed in the vicinity of Purkinje cell bodies at about P13. They were later found throughout the cerebellum of Grid2^*Lc/+*^ adult mice, particularly in the vicinity of blood vessels (Figure 1D). In the adult wild-type cerebellum (Figure 1C) and in the other brain structures, such as the brainstem, the microglia remained non-activated (data not shown).

**Figure 2:**
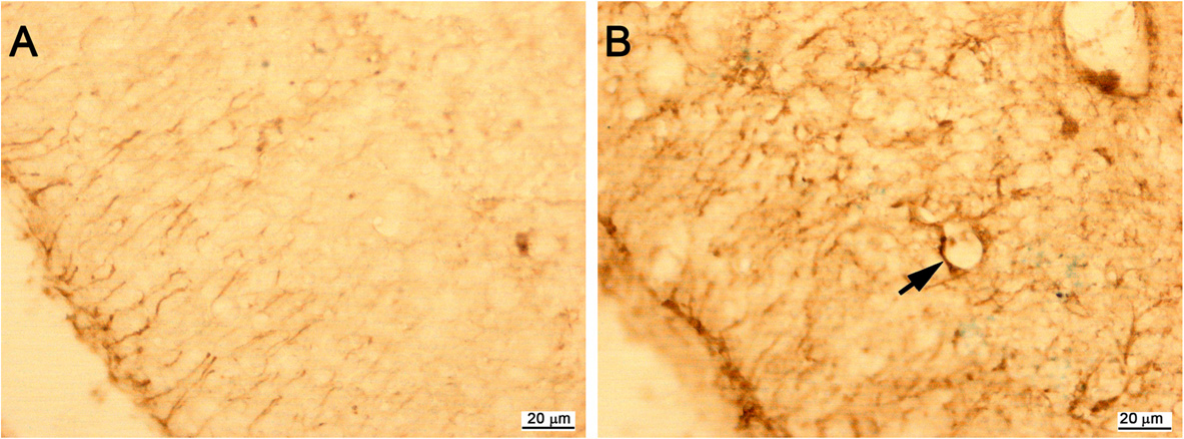
**Astrocyte activation in the Grid2**^***Lc/+ ***^**cerebellum. **Sagittal sections of wild-type **(A) **and Grid2^*Lc/+ *^**(B) **cerebella from 21-day-old mice were immunolabeled for glial fibrillary acidic protein. Strongly stained astrocytes have colonized the degenerating granular layer of the Grid2^*Lc/+ *^cerebellum and surround blood vessels with their enlarged processes (arrows). Scale bars: 20 μm.

**Figure 3:**
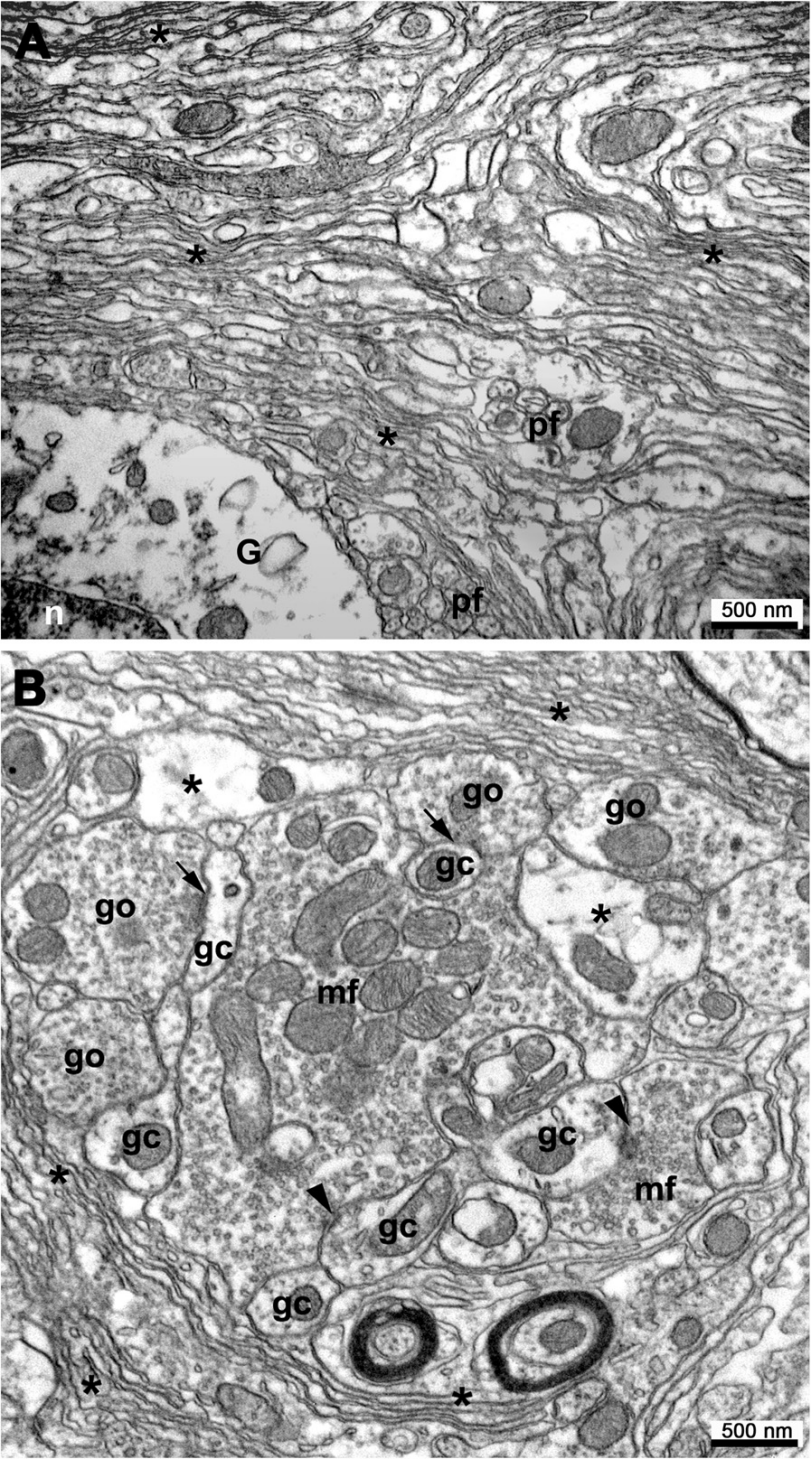
**Astrogliosis in the adult Grid2**^***Lc/+ ***^**cerebellum. **Representative electron microscopy views of the molecular layer **(A) **and of the internal granular layer **(B)**. In the molecular layer, a few surviving parallel fibers (pf) are wrapped in abnormally abundant, superimposed astrocytic processes (asterisk); G, glial cell soma with electrolucent cytoplasm; n, nucleus. In the internal granular layer, abundant astrocytic processes are stacked around a cerebellar glomerulus made of a mossy fiber-like terminal (mf) forming asymmetric synapses (arrowheads) on a few granule cell dendrites (gc), which receive inputs from Golgi neuron terminals (go) at symmetric synapses (arrows). Bar: 500 nm.

Paralleling the microglial reaction, astrocytes displayed changes in morphology from P13 and P21 into adulthood. In the P21 degenerating Grid2^*Lc/+*^ cerebellum, fibers of Bergmann glia were shorter and less regularly spaced than in the cerebella of control littermates (data not shown). The granular layer was invaded by numerous strongly stained astrocytes, which persisted during adulthood (Figure 2B). Electron microscopy showed that all surviving neuronal processes - a few parallel fibers, basket and stellate interneurons in the molecular layer and rare glomeruli in the internal granular layer - were rolled up into hyperdeveloped, superimposed astrocytic processes extending throughout the atrophied cerebellar cortex of the Grid2^*Lc/+*^ mutants (Figures 3A,B).

### Long-term astrocyte activation is preceded by acute *gfap* gene activation

**Figure 4:**
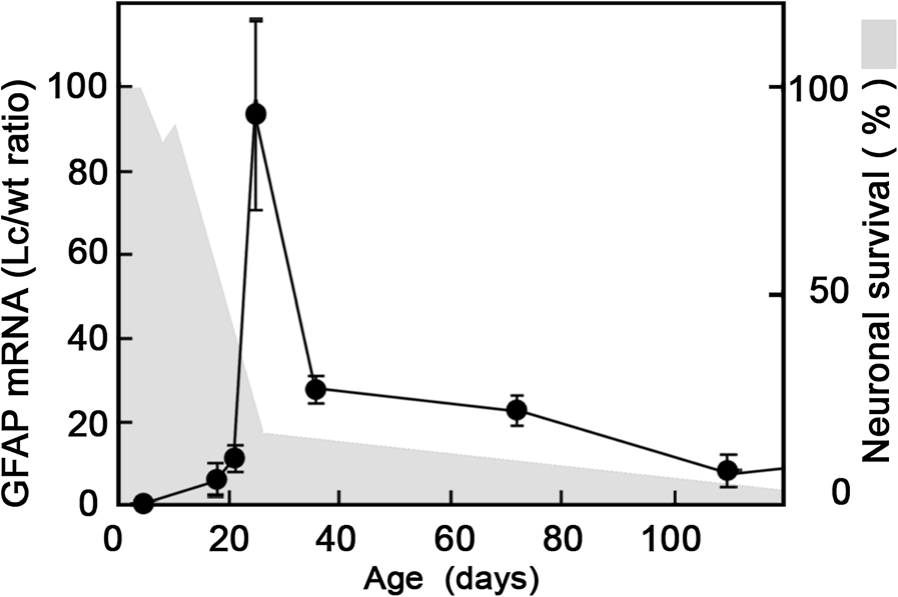
**Time course of *gfap *mRNA levels in the Grid2**^***Lc/+ ***^**(Lc) cerebellum from postnatal day 5 (P5) to P110. ***Gfap *mRNA is present in large amounts from P22 to P120. The *gfap *mRNA is produced in a large burst coinciding with the end of the episode of massive neuron degeneration. The results are expressed as the ratio of Grid2^*Lc/+ *^*gfap *mRNA to wild-type *gfap *mRNA, and the means ± SEM of four to six paired age-matched Grid2^*Lc/+ *^and wild-type mice are shown. Age-related survival of neurons (shaded in gray), including Purkinje and granule cells, was determined from the estimates of Caddy and Biscoe [11]. Survival is expressed as a percentage of the number of cerebellar neurons on P4.

GFAP upregulation is a hallmark of astrocyte activation. We investigated the transcriptional regulation of GFAP in the Grid2^*Lc/+*^ cerebellum by analyzing *gfap* mRNA levels at various time points in the Grid2^*Lc/+*^ and wild-type cerebellum (Figure 4). In the wild-type cerebellum, *gfap* mRNA levels decreased from P5 to P18 and remained stable and low throughout the rest of the study period (data not shown). In the Grid2^*Lc/+*^ cerebellum, *gfap* mRNA levels increased strongly between P22 and P25, peaking sharply at a level 93 times higher than that in the wild type. In parallel to the age-related neuron survival, according to the estimates reported by Caddy and Biscoe for the Lurcher cerebellar neuron population [11], a burst of *gfap* mRNA production was found to occur at the end of a period of extensive neuron (Purkinje and granule cell) loss. From P36 until P110 *gfap* mRNA levels remained high. They stabilized in adult Grid2^*Lc/+*^ cerebellum at a level 10 times that in wild-type cerebellum.

### Increases in CD95 expression reflect astroglial cell activation

**Figure 5:**
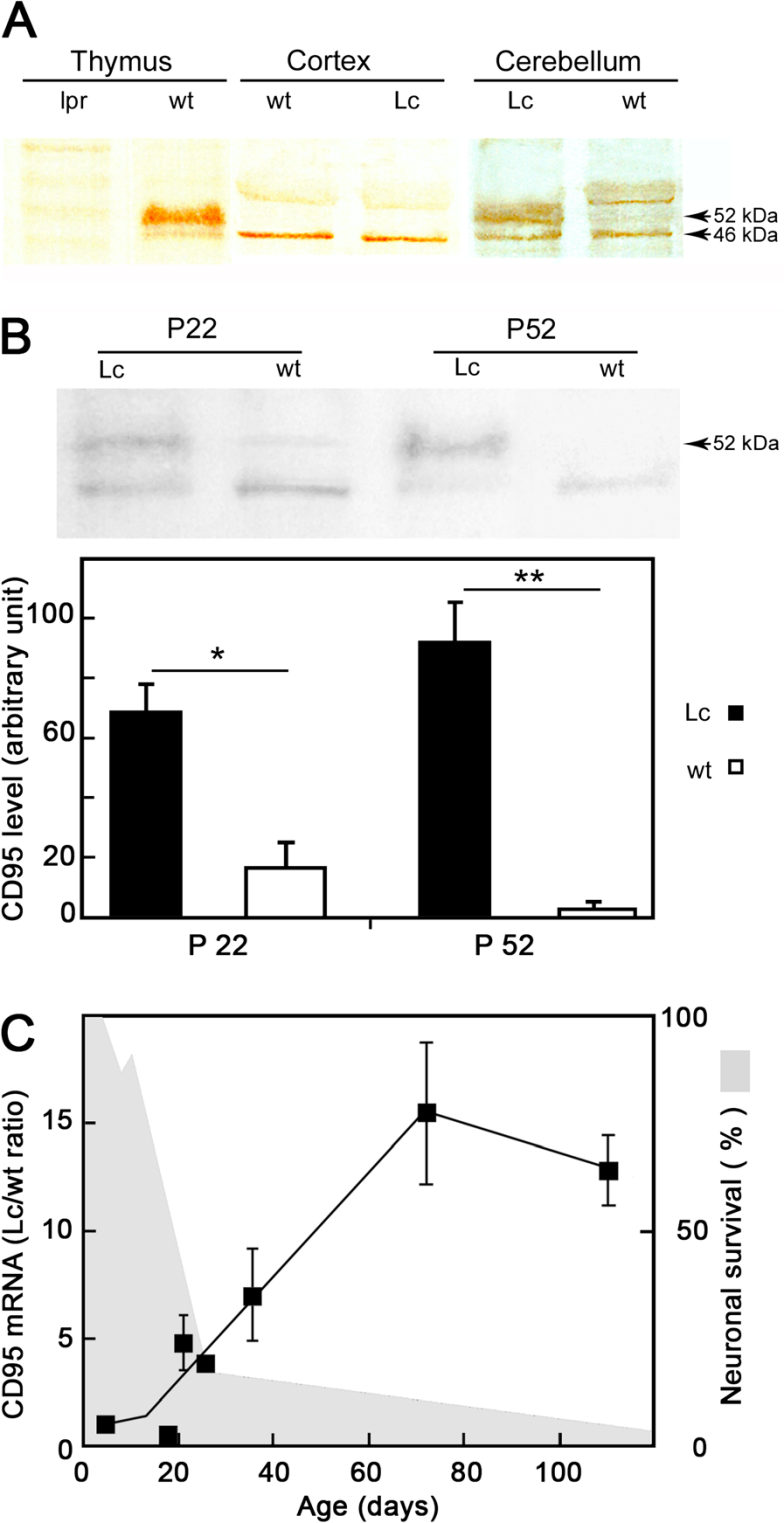
**CD95 expression in the Grid2**^***Lc/+ ***^**cerebellum. (A) **Western blot analysis of the CD95 protein in crude extracts of cerebral cortex and cerebellum from Grid2^*Lc/+ *^(Lc) mutant and wild-type (wt) mice. Thymus extracts from *lpr *and wt mice were used as negative and positive controls, respectively. **(B) **CD95 western blot analysis at two time points - postnatal day 22 (P22) and P52 - in wt and Lc cerebella, and relative densitometric quantification of the 52 kDa band, expressed as a percentage of a reference protein sample. Values are the means ± SEM of four independent determinations (one-way analysis of variance followed by de Sheffé multiple comparison test, *P <0.05, **P <0.01). **(C) **Time course of CD95 mRNA production in the cerebellum of Lc and wt mice from P5 to adulthood. The results are expressed as the ratio of the Lc CD95 mRNA to wt CD95 mRNA, and the means ± SEM of four paired age-matched Lc and wt mice are shown. Note that CD95 levels begin to increase at the end of the episode of massive neuron degeneration (shaded in gray; see Figure 4).

CD95 is expressed principally on neurons and, to a lesser extent, on glial cells in the adult brain [2]. Its expression is upregulated in pathological conditions. We investigated the contribution of CD95 to Grid2^*Lc/+*^ cerebellar degeneration by studying CD95 expression in the cerebellum of Grid2^*Lc/+*^ and wild-type mice by western blot analyses of crude cerebellar extracts (Figure 5). The anti-CD95 antibody used cross-reacts with undefined proteins, as previously described [20]. We thus included a negative control in our study: thymic extracts from *lpr* mice, which have only very low levels of CD95 [21]. CD95 was identified as a 52 kDa band. A 46 kDa band was consistently detected in all tested brain tissue extracts and may correspond to a non-glycosylated form of CD95 (Figure 5A) [20]. We evaluated CD95 expression in the Grid2^*Lc/+*^ mutant and wild-type littermate cerebella at P22 and P52. P22 corresponds to a period of massive neuron loss. One month later, at P52, the neuronal death process is coming to an end [11]. CD95 was significantly more strongly expressed in the Grid2^*Lc/+*^ than in the wild-type cerebellum, and its expression increased steadily from P22 to P52 (Figure 5B).

We characterized CD95 upregulation in the Grid2^*Lc/+*^ cerebellum further, by studying CD95 transcripts from P5 to P110. In the Grid2^*Lc/+*^ cerebellum, transcripts levels were similar to those in the wild type until P18. From P18 onwards, they increased over a period of 8 weeks, eventually reaching a plateau at a level 10 times that in the wild type (Figure 5C). CD95 mRNA levels were still high in 16-month-old Grid2^*Lc/+*^ mice (data not shown).

Thus, the increase in CD95 expression was due to an increase in the number of CD95 transcripts, indicating the involvement of a transcriptional mechanism.

**Figure 6:**
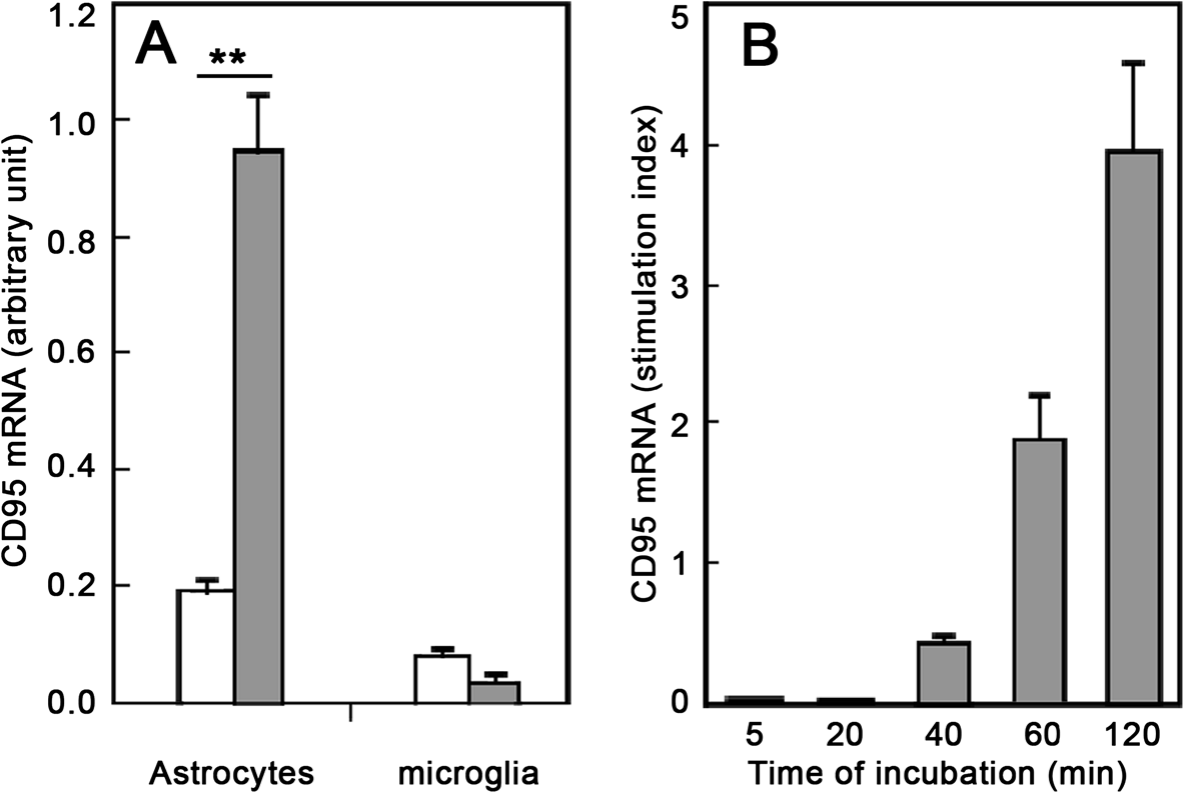
**CD95 mRNA levels in cultured astrocytes. (A) **Cultures highly enriched in astrocytes and microglia were stimulated with a mixture of IL-1β (20 ng/ml) and TNFα (50 ng/ml) for 2 hours (gray bars). Open bars, unstimulated cell culture. **(B)** Kinetic study of CD95 mRNA production in stimulated astrocyte cultures. The values shown are the means ± SEM of four different glial cell cultures (Mann Whitney test, **P <0.01).

We investigated the cellular distribution of CD95 by carrying out immunohistochemistry studies in brain slices with several anti-CD95 antibodies. Studies of CD95-deficient mice, used as a negative control, showed that none of the antibodies tested, including those used for western blotting, was entirely specific for CD95 in brain slices, as previously reported [22]. Thus, we looked for CD95 transcripts in cultures highly enriched in wild-type and Grid2^*Lc/+*^ astrocytes and microglia (Figure 6). No difference was found between wild-type and mutant cells. Non-stimulated resting astrocytes expressed CD95, whereas microglia did not. CD95 transcript levels were upregulated by proinflammatory treatment in astrocytes, but not in microglia, in which neither lipopolysaccharide (LPS) nor proinflammatory cytokines (data not shown) induced CD95 expression (Figure 6A). In cultured astrocytes, high levels of CD95 were detected after 40 minutes of stimulation with proinflammatory cytokines, these levels increasing for up to 2 hours (Figure 6B).

### Upregulation of soluble CD95L and inflammatory cytokines

**Figure 7:**
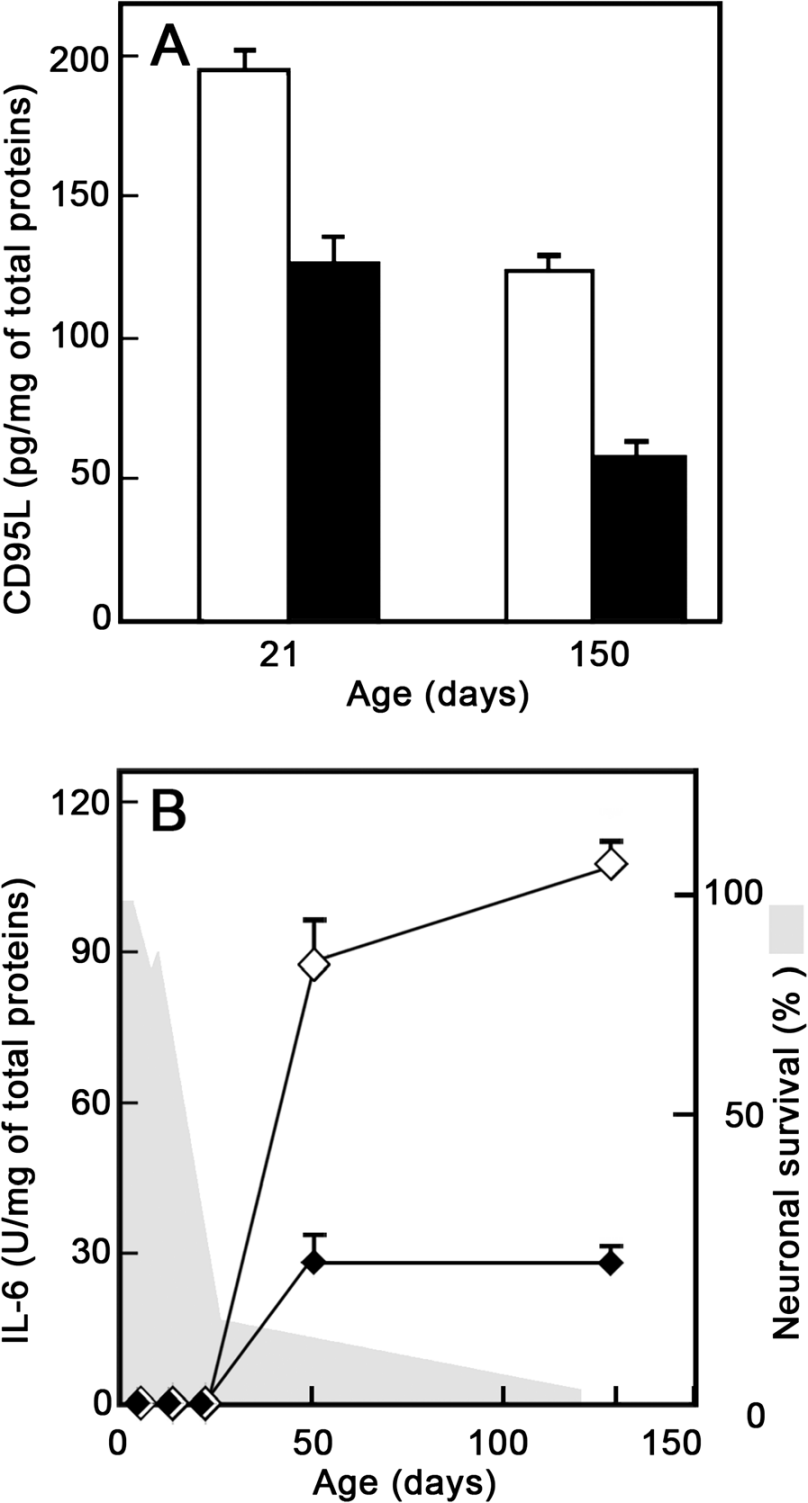
**CD95L and IL-6 levels in the cerebellum of Grid2**^***Lc/+***^**. (A) **Representative experiment from the CD95L study. CD95L concentrations were determined in crude cerebellum extracts from Grid2^*Lc/+ *^(open bars) and wild-type (black bars) mice, by ELISA. **(B) **Representative time-course study of IL-6 levels in the cerebellum of Grid2^*Lc/+ *^(open diamonds) and wild-type (black diamonds) mice. Levels were determined in a biological activity assay. Note that IL-6 levels begin to increase at the end of the period of massive neuron degeneration (shaded in gray; see Figure 4). The mean values for four determinations are shown, with range error bars.

In normal brain, inflammatory cytokines are either absent or are produced in quantities only just exceeding the detection threshold. These molecules are induced or upregulated during brain inflammation featuring glial cell activation. We previously showed that levels of the proinflammatory cytokine IL-1ß were higher in 4-month-old Grid2^*Lc/+*^ mice than in the wild type [17]. We investigated the involvement of CD95L in the glial reaction in Grid2^*Lc/+*^ cerebellum by studying soluble CD95L levels in wild-type and mutant mouse cerebellum. Determinations were carried out at two time points - during the period of intense degeneration and much later, at P21 and P150, respectively (Figure 7A). CD95L was detected in the cerebellum of young wild-type mice, but it increased in abundance with age. CD95L levels in the Grid2^*Lc/+*^ cerebellum were higher than those in the wild type at both ages. In the cerebral cortex of mutant and wild-type mice, CD95L levels were in the range of values found for the P150 wild-type cerebellum (data not shown). We also investigated changes in the amount of IL-6, an inflammatory cytokine produced mostly by astrocytes and, to a lesser extent, microglia, during brain inflammation. We determined IL-6 levels during and after neurodegeneration, from P5 to P130 (Figure 7B). IL-6 was undetectable during the first 3 weeks after birth in both wild-type and Grid2^*Lc/+*^ cerebella. From P50 to adulthood, IL-6 was present at stable, low levels in the cerebellum of the wild-type mice but at levels three times higher in Grid2^*Lc/+*^ mice.

## Discussion

The CD95 receptor and its ligand, CD95L, are thought to contribute to brain neural death and inflammation processes through non-apoptotic signaling. In this study, we investigated the role of the CD95/CD95L system in the cerebellum of the Grid2^*Lc/*+^ mutant mouse, a model of naturally occurring brain parenchyma neurodegeneration without damage to the blood–brain barrier. We focused on the associated inflammatory reaction. We studied glial cell activation and CD95 expression before, during and after cerebellar neuron loss. Levels of CD95 and its ligand were high in mutant mice, and we observed a protracted inflammatory reaction triggered by the early onset of cerebellar neuron death. The correlations identified in studies of the time courses of CD95 expression, neuron survival and astrocyte activation indicated that CD95 was expressed on glial cells, but not on neurons, and might therefore contribute exclusively to the inflammatory process.

The time course and mechanisms of death of the various types of neurons in mutant mice have been widely studied and are well understood [8]. In this study, we studied the glial reaction to neuron loss. Analyses of the morphological and biochemical criteria for glial cell activation showed that astrocytes and microglia were activated in a process characterized by an early onset and a burst of activation between P22 and P25, coinciding with peak levels of neuron cell death. From P7 to P30, 90% of the Purkinje cells and granular neurons disappeared from the Grid2^*Lc/+*^ cerebellum. Purkinje cells genetically committed to death display morphological and physiological alterations that can be detected as early as P6 and they die between P10 and P30 [11]. Our findings, consistent with the kinetics of Purkinje cell and granular neuron degeneration, show that microglia are activated after P5 and before P13 and that not all microglial cells are reactive at P13, probably due to the gradual loss of cerebellar neurons. Furthermore, the clusters of microglia found in a few areas of the Purkinje cell layer were consistent with the “holes” lacking Purkinje cells in this layer described in P12 Grid2^*Lc/+*^ by Dumesnil-Bousez and Sotelo [23]. Clustered microglia are probably of the macrophage type, playing a role in the removal of dead cells [24,25]. Complete microglial activation throughout the cerebellum occurs during the period of mass neuron death between P17 and P25 [11]. Astrocyte activation parallels microglial activation, but with a small time lag, in the stereotypical glial reaction to brain injury. Microglial cells are fully activated at about P25 to P26, whereas astrocytes are only just beginning to be activated at this time point, as shown by the upregulation of GFAP. Astrocyte activation begins with a huge burst of *gfap* transcription (resulting in transcript levels 93 times higher than those in the wild type) coinciding with a period of mass neuron death in the Grid2^*Lc/+*^ cerebellum. In the adult Grid2^*Lc/+*^ cerebellum, *gfap* mRNA levels remain 10 times higher than those in the wild type, and microglial clusters may persist for up to 5 months. Electron microscopy revealed that, throughout the highly disturbed cytoarchitecture of the Grid2^*Lc/+*^ cerebellum, astrocytes ensheathed the surviving neurons - basket, stellate and Golgi cells [11] - in a large number of stacked astrocytic processes forming a cocoon-like structure.

The Grid2^*Lc/+*^ glial response resembles experimental models of acute neuronal degeneration, in which dead neurons are rapidly removed by phagocytic microglia, and neuron-depleted areas are filled with astrocytes, leading to the formation of a scar [25,26]. Nevertheless, it differs from these models in the persistence of activated microglial cells long after the neurodegenerative event. For example, in the mouse model of kainic acid-induced hippocampal neuronal death, for doses of kainic acid destroying all pyramidal cells in the injected hippocampus without affecting non-neuronal cells, the number of microglia with a quiescent cell morphology returns to normal after about 3 months [27]. Thus, the Grid2^*Lc/+*^ microglial reaction triggered by early neuron loss seems to decline very slowly, taking a particularly long time to come to an end.

In the Grid2^*Lc/+*^ cerebellum, the onset of the neuron loss takes place early after birth, overlapping a period known to have specific inflammatory properties. Lawson and Perry [28] have shown that, during postnatal development, there is a window of susceptibility to induce a classical inflammatory response, resembling that seen in non-CNS tissues. They showed that, in contrast to adult mice, 7-day-old mice injected with LPS in the parenchyme develop a vigorous inflammatory response with prolific neutrophil recruitment and a brisk mononuclear phagocyte response. In contrast to the model of Lawson and Perry, Grid2^*Lc/+*^ glial cell activation occurs without leakage of the blood–brain barrier and peripheral cell intrusion (N Delhaye-Bouchaud and J Mariani, unpublished data). Thus, the glial activation intensity may be related to the age-related properties of the inflammatory response while the absence of infiltrating peripheral cells, known to contribute to the healing mechanisms [29], may be responsible for the persistence of the glial reaction. Taken together, this may give an unusual dynamic to the glial reaction in the Grid2^*Lc/+*^ cerebellum.

The cellular origin of CD95 in the brain has been the subject of lively debate. Neurons, astrocytes and oligodendrocytes have all been shown to express CD95, and it is now thought that neurons are the strongest expressers of CD95 in the normal adult brain, whereas CD95 expression is detected on glial cells in pathological conditions. CD95L is present in the body as a membrane-bound protein mediating apoptotic signaling, or as a metalloprotease-cleaved soluble form. In the brain, CD95L has been reported to be constitutively expressed on neurons and astrocytes and upregulated by inflammation [2,30].

In the Grid2^*Lc/+*^ cerebellum, increases in the levels of both GFAP and CD95, and decreases in neuron survival occur simultaneously, suggesting that the CD95 synthesized *de novo* originates from astrocytes. Unfortunately, we were unable to show that CD95 was located on astrocytes by immunohistochemical methods, due to the known lack of specificity of antibodies directed against the mouse protein [22]. However, increase in CD95 levels in response to stimulation on cultured astrocytes [31,32] but not on microglia suggests that the newly synthesized CD95 is of astrocytic origin. The soluble form of CD95L (sCD95L) is also abnormally expressed in the Grid2^*Lc/+*^ cerebellum. Peripheral cells, such as T cells or cells of the monocyte lineage, cannot be held responsible for the production of CD95L in this context, because peripheral immune cells are not recruited to the brain during cerebellar degeneration in Grid2^*Lc/+*^ mice (N Delhaye-Bouchaud and J Mariani, unpublished data). However, the CD95L producing cells within the Grid2^*Lc/+*^ cerebellum remain to be identified.

In the Grid2^*Lc/+*^ cerebellar inflammatory reaction, CD95L might be involved in several of the mechanisms of reactive gliosis. Recently, sCD95L has been implicated in cell migration via a non-apoptotic pathway. The underlying mechanism involves the formation of a specific signaling complex containing CD95 [30]. Cell migration is an important process in the cascade of events occurring during the inflammatory reaction, and is a feature of microglial cells in the CNS. Several mechanisms involving different microglial attractants have been described [33] and it is not possible to rule out the possibility that sCD95L belongs to the microglial motility machinery. However, sCD95L might play a role in the motility of the astrocytes, which grow and fill the spaces vacated by the lost neurons. Less speculative is the role of CD95 activation in the induction or upregulation of inflammatory mediators, such as IL-1, IL-6, IL-8 and cell surface integrins [34]. We found that IL-6 concentration increased with glial cell activation. In a previous study, abnormally high basal levels of IL-1ß were detected in adult Grid2^*Lc/+*^ cerebellum [17]. These two cytokines play an essential role in the inflammatory cascade. IL-6, which is mostly produced by astrocytes during brain inflammation, triggers astrocyte proliferation and is believed to be involved in astrogliosis and the activation of microglia [31,35]. Microglia are the principal inducible source of IL-1ß, a strong inducer of astrocyte proliferation [36]. Thus, within the adult Grid2^*Lc/+*^ cerebellum, reactive gliosis may self-perpetuate for some time through the cytokine and chemokine network, including the CD95/CD95L system, in the absence of infiltrating peripheral immune cells. Such peripheral immune cells play a key role in the repair cascade resulting in the healing of brain wound [29].

In conclusion, our findings support the involvement of the CD95/CD95L system in the glial reaction triggered by neuron death, through non-apoptotic signaling in the neurodegenerative cerebellum of Grid2^*Lc/+*^ mutant mice. The persistence of microglial activation in this model is consistent with the misregulation of molecular mechanisms responsible for the termination of glial activation, in which the role of the CD95/CD95L system remains to be determined.

## Abbreviations

AEBSF: 4-(2-Aminoethyl) benzenesulfonyl fluoride hydrochloride; CNS: central nervous system; EDTA: ethylenediaminetetraacetic acid; ELISA: enzyme-linked immunosorbent assay; GFAP: glial fibrillary acidic protein; HPRT: hypoxanthine-guanine phosphoribosyltransferase; IL: interleukin; PX: postnatal day X; LPS: lipopolysaccharide; RT-PCR: reverse transcriptase polymerase chain reaction; SBTI: soybean trypsin inhibitor; sCD95L: soluble CD95L; SI: stimulation index; TBS: Tris-buffered saline.

## Competing interest

The authors declare that they have no competing interests.

## Authors’ contributions

BVDG conceived the project and designed the study, carried out the molecular and biochemical studies and wrote the manuscript. PD carried out the light microscopy studies.^.^ YB carried out the electron microscopy study. JM helped with project conception and with critical analyses of the results and revision of the manuscript. All authors have read and approved the final manuscript.
